# Development of an Efficient In Vitro Propagation Method for *Acmella oleracea* (L.) R.K. Jansen

**DOI:** 10.3390/mps9020044

**Published:** 2026-03-08

**Authors:** Pál Szarvas, Judit Csabai, Anzhela Kolesnyk, Judit Dobránszki

**Affiliations:** 1 Centre for Agricultural Genomics and Biotechnology, Faculty of the Agricultural and Food Science and Environmental Management, University of Debrecen, P.O. Box 12, H-4400 Nyíregyháza, Hungary; szarvas.pal@agr.unideb.hu; 2 Institute of Engineering and Agricultural Sciences, University of Nyíregyháza, Sóstói Str. 31/b, H-4400 Nyíregyháza, Hungary; 3 Department of Genetics, Plant Biology and Microbiology, Uzhhorod National University, 3 Narodna Square, 88000 Uzhhorod, Ukraine; angela.kolesnyk@uzhnu.edu.ua

**Keywords:** *Acmella oleracea*, carbon source, cytokinins, disinfection, in vitro culture, micropropagation, rooting, shoot multiplication, tissue culture

## Abstract

*Acmella oleracea* (L.) R.K. Jansen is an herbaceous plant cultivated globally as an annual ornamental species. While conventional propagation methods exist, the lack of a standardized in vitro protocol limits research and industrial applications that require genetically and morphologically uniform plant material. In this study, in vitro cultures of *A. oleracea* were established via seed germination. Well-developed in vitro shoots were dissected into individual nodal segments to serve as explants. Multiple media were evaluated for regeneration and growth, including full-, half, and quarter-strength Murashige and Skoog (MS) media, as well as full- and half-strength McCown Woody Plant media. Two carbohydrate sources, saccharose and glucose, were tested at concentrations of 1%, 2%, and 3% (*w*/*v*) in the multiplication medium. Subsequently, the effects of different cytokinins were assessed at concentrations of 4.4 µM and 13.2 µM. The findings demonstrated that 13.2 µM meta-Topolin with 3% saccharose, or 13.2 µM Benzyladenine with 2% glucose was most beneficial for shoot multiplication of *A. oleracea*. The multiplied shoots were rooted in vitro within 13 days, then potted and acclimatized within 8 days. This protocol facilitates future industrial applications of *A. oleracea*, particularly in the cosmetics sector, where the use of standardized biomass is essential.

## 1. Introduction

*Acmella oleracea* (L.) R.K. Jansen is an herbaceous annual plant from the *Asteraceae* family, known for its unique phytochemical profile and expanding applications in cosmetic and pharmaceutical formulations. Currently accepted as the valid scientific name in major taxonomic databases, *Acmella oleracea* is the most widely cultivated among the 36 recognized species of the genus. It has five homotypic and eight heterotypic synonyms; The Plant List reports 11 synonyms, reflecting its complex taxonomic history [1,2].

Commonly referred to as the “toothache plant,” “jambu,” or “electric daisy,” this species is native to South America but is now cultivated globally. It is a low-growing, soft-stemmed, branched annual herb that reaches heights of 20–60 cm. The leaves are ovate to lanceolate with serrated margins, and the green, slightly ribbed stem supports discoid capitula composed of tubular yellow florets with a central red disk. The fibrous root system provides anchorage and nutrient absorption [3,4,5]. Natural populations exhibit high morphological and genetic variability, which poses challenges for standardization but also presents opportunities for selecting elite lines for targeted applications [6].

The leaves have a strong, tingling, or numbing flavor due to the main compound, spilanthol [3,4,5]. The concentrated extract of plant parts (especially inflorescences or roots), known as jambu extract or jambu oil, can be used as a flavoring agent or medicinal preparation. Studies have shown that spilanthol exhibits anti-inflammatory, antimicrobial, antipyretic, antioxidant, cardioprotective, insecticidal, antiseptic, immune-stimulatory, anti-obesity, anticancer, antifungal, diuretic, larvicidal, and antioxidant effects [4,7,8,9,10,11,12,13]. Jahan et al. [14] examined the effects of stem extracts and in vitro-derived callus against various bacteria. Besides spilanthol, the plant also contains acmellonate, scopoletin, phytosterols, terpenes, esters, and aromatic, phenolic, flavonoid, and coumarin compounds [4,5,15]. Bioactive compounds are also synthesized by in vitro-cultured plantlets [16].

*A. oleracea*, also known as the “natural or herbal Botox” [17], is gaining popularity in cosmetics because of its main bioactive compound, which has anesthetic [18], muscle-relaxant, and mild neuro-sensory effects (Malik et al., 2025) [19]. Recent studies also indicate that spilanthol can regulate muscle tone and improve circulation [20]. These qualities make it suitable for products aimed at wrinkle reduction and anti-aging [21]. Savi et al. confirmed that an *A. oleracea* extract-loaded glycolipid-based serum is stable, exhibits preliminary safety (no ocular irritation, no dermal absorption, and good skin tolerability), and is effective (improving skin hydration and microrelief), supporting its potential as a safe and effective topical anti-aging treatment [22].

This plant is widely distributed globally, was originally identified in Peru, and is commonly found in tropical and subtropical regions. It is cultivated worldwide as an annual ornamental plant because it is highly frost-sensitive [7,23]. As an annual plant, it is propagated by seeds, although seed viability declines rapidly [8,24]. Its natural ability to root from nodal parts is limited, making vegetative propagation difficult [8,24].

In natural habitats, both abiotic and biotic factors influence plant growth and the accumulation of bioactive compounds. Such factors include seasonal variations, the presence of pathogens or pests, soil nutrient content, and the quality of land available for cultivation. For industrial purposes, a continuous supply of bioactive compounds is required. In vitro propagation provides an efficient alternative for producing large quantities of selected plant material under controlled conditions, ensuring consistently high levels of active ingredients and offering opportunities for genetic resource conservation [8,9,15,24].

Previous in vitro propagation studies of *A. oleracea* have predominantly used shoot tips or nodal segments derived from in vivo-grown plants. Algabri and Pandhure [24] cultured shoot segments on half-strength MS medium [25] supplemented with BA, IAA, and KIN, and reported the highest multiplication rate of 4.3 shoots per explant with 1 mg L^−1^ BA. However, the percentage of responding explants was only 35%, meaning that the shoot number converted to all starting explants reached only 1.5. Mohan et al. [15] developed an in vitro flowering protocol using BA, KIN, IBA, and AgNO_3_, achieving 65.2% flowering with a combination of 1.0 mg L^−1^ KIN, 0.5 mg L^−1^ IBA, and 5.0 mg L^−1^ AgNO_3_. Nabi et al. [26] evaluated various combinations of plant growth regulators (PGRs) for both shoot and callus induction. The optimal shoot regeneration from nodal segments was achieved with 0.5 mg L^−1^ BA and 0.5 mg L^−1^ IBA, while callogenesis from leaf explants was most effective with 1 mg L^−1^ 2,4-D, 0.5 mg L^−1^ KIN, and 0.5 mg L^−1^ BA, with a 95.5% success rate. The highest shoot regeneration frequency (90.5%) was obtained on medium containing BA in combination with IBA at 0.5 mg L^−1^ each. The maximum mean shoot number was 8.5, with a 7.3 mean shoot length. The highest root regeneration rate ranged from 70% to 90%. Sharma and Shahzad [27] induced shoot buds (1 cm with a pair of leaf primordia) from shoot tips of seedlings using 0.25 µM TDZ, followed by shoot multiplication in 1 µM BA and rooting in 2.5 µM NAA. The explant response rate was 98%, with a mean of 30 shoots per explant. Lima et al. [28] initiated cultures from *A. oleracea* seeds and observed enhanced germination with BA or KIN in the presence of 3% saccharose and 0.05 g L^−1^ ascorbic acid. The highest germination percentage (65%) was observed on medium containing 0.1 mg L^−1^ KIN, while the germination reached only 56% on PGR-free medium. Joshi et al. [29] tested the effect of agar–agar concentration and the liquid medium on the shoot multiplication capability of Acmella. Multiplication rates increased as the agar–agar concentration was reduced (0.8% < 0.5% < 0.2% < 0%). The highest shoot number per explant (6.2) was obtained on liquid MS medium containing 1 mg L^−1^ KIN. Although rooting occurred spontaneously, it was further optimized with 1 mg L^−1^ IBA, resulting in 100% survival after acclimatization.

In addition to *A. oleracea*, several studies have examined in vitro propagation of related *Acmella* species. Rios-Chavez et al. [30] developed a hormone-free regeneration protocol for *A. radicans*. Senthilkumar et al. [31] and Razaq et al. [32] investigated *A. calva*, using conventional cytokinins and auxins to induce callogenesis or shoot formation.

Although studies demonstrating the micropropagation potential of *A. oleracea* most employed a limited range of media and growth regulator combinations, none employed systematic, multifactorial experimental designs. Key variables such as media type, carbon source, and cytokinin variations were either fixed or investigated only minimally.

The cosmetics industry is increasingly seeking bioactive ingredients that are both natural and effective, with spilanthol emerging as a candidate for anti-aging and wrinkle-reducing formulations. To meet this demand, propagation methods must produce uniform, contaminant-free, and metabolically consistent plant material. In vitro maintenance and multiplication offer key benefits, including metabolite profiling, extract quality control, and preservation of genetic resources, regardless of environmental variability (Kulak et al., 2022) [33].

This study addressed these requirements by systematically testing a range of culture conditions, such as basal media (MS, Mc Cown Woody Plant (MWP) [34], and their half-strength versions), carbohydrate sources (saccharose and glucose at 1–3%), five cytokinins (BA, TDZ, Zeatin, meta-Topolin, and 2iP), and rooting treatments with or without NAA, and by evaluating their effects on regeneration and acclimatization performance.

## 2. Materials and Methods

### 2.1. Seed Germination and Establishment of In Vitro Cultures

At first, the germination ability of seeds was tested on wetted, sterilized filter paper in closed Petri dishes, without prior surface disinfection, under light conditions in a culture room. In total, 100 pieces of seeds were tested in this experiment.

Next, two procedures were tested for seed surface disinfection (Table 1):

(1) Seeds were washed in Ultra Sol™ for 5 min, then surface sterilized in a 0.1% HgCl_2_ solution with one drop of Tween-80 for 4 min. They were rinsed in sterile deionized water for 1 min, followed by treatment with 70% ethanol for 30 s, and then soaked in deionized water for 10 min. This soaking step was repeated three times. A similar method was used by Kumar et al. [15], employing the same HgCl_2_ concentration. In total, 156 seeds were surface disinfected in this experiment.

(2) Seeds were washed in Ultra Sol™ for 5 min, then sterilized in 1/3-strength commercial sodium hypochlorite (NaOCl) solution with one drop of Tween-80 for 6 min, and rinsed in sterile deionized water (SDW) for 1 min. Afterward, they were treated with 70% ethanol for 30 s and soaked in deionized water for 10 min; this final soaking step was also repeated three times. Sodium hypochlorite was used as a surface disinfectant in a method described by Lima et al. [28]. In total, 156 seeds were surface disinfected in this experiment.

The germination of seeds was performed in vitro on half-strength MS medium supplemented with 2% saccharose, following surface disinfection.

Well-grown shoots of *A. oleracea* were dissected into single nodal segments [15,30]. Shoot tips and nodal segments were cultured on full-strength MS medium, enriched with 3% (*w*/*v*) saccharose, and solidified with 6.5 g L^−1^ agar, without any plant growth regulators (PGRs). The pH was adjusted to 5.7 prior to autoclaving. All media were sterilized by autoclaving at 121 °C and 1.2 bar for 20 min.

General culture conditions were maintained at 23 ± 2 °C with a 16/8 h photoperiod provided by a 1:1 mix of daylight and warm-white fluorescent tubes. All cultures were grown in 370 mL jars filled with 70 mL of MS medium (pH 5.7–5.8, sterilized at 121 °C, 1.2 bar for 20 min). Light intensity was specifically adjusted to the developmental stage: 25–35 µmol m^−2^ s^−1^ was applied during seed germination (on half-strength MS with 2% saccharose) to avoid photoinhibition, while 80–106 µmol m^−2^ s^−1^ was used for the subsequent multiplication and rooting phases (on full-strength MS with 3% saccharose and 6.5 g L^−1^ agar).

To obtain sufficient plant material for the experiments, shoot multiplication was performed on PGR-free full MS medium with 3% saccharose using single-node segments.

### 2.2. Investigation of Minerals of the Shoot Multiplication Medium

Various media types and strengths were tested to maintain *A. oleracea* shoots. Full-, half-, and quarter-strength MS macro salts, as well as full- and half-strength MWP macro salts, were applied as micropropagation media, without any PGRs. Microelements and vitamins were applied at full strength in all treatments. All culture media contained 3% saccharose. Single-node segments were used as explants. Five explants were placed vertically in each vessel. A total of 90–100 explants per treatment were used, cultured in 18–20 vessels. The total number of explants used in this experiment was 485.

The following parameters were recorded:

- number of shoots (SN);
- length of shoots (SL, in mm);
- number of nodes per shoot (NN);
- number of leaves per shoot (LN);
- root length per shoot (RL, in mm);
- number of roots per shoot (RN).

Additionally, the following ratios were assessed:

- callus formation rate: (number of explants producing callus/total number of explants) × 100;
- explants exhibiting hyperhydration: (number of hyperhydrated explants/total number of explants) × 100;
- shoot regeneration rate: (number of explants regenerating shoots/total number of explants) × 100;
- simultaneous shoot and callus formation: (number of explants producing both shoots and callus/number of explants regenerating shoots) × 100;
- hyperhydricity rate of regenerated shoots: (number of shoots showing hyperhydration symptoms/total number of regenerated shoots) × 100.

The experiment lasted for four weeks.

### 2.3. Investigation of the Carbon Source of the Shoot Multiplication Medium

A total of 1%, 2%, or 3% of saccharose or glucose was added as a carbohydrate source to full-strength MS culture medium. One-node segments from shoots were used as explants. Five explants were placed vertically in each vessel. A total of 90 explants per treatment were cultured in 18 vessels, resulting in 540 explants in total for this experiment. The following growth parameters were recorded: SL, SN, RL, RN, NN, LN, SW, RW, as well as callus formation and hyperhydricity.

The experiment lasted for four weeks.

### 2.4. Effects of Various Cytokinins Applied in the Shoot Multiplication Medium

Based on the results obtained during the carbohydrate optimization phase (Section 2.3), 2% glucose and 3% sucrose were selected as the optimal concentrations and were used in all subsequent experiments involving cytokinin treatments. The following cytokinins were tested in two concentrations (CK-1: 4.4 µM, CK-2: 13.2 µM): BA [15,24,26,29], Zeatin (ZEA), N6-(2-Isopentenyl)adenine (2iP), meta-Topolin (MTP), and Thidiazuron (TDZ) [27]. One-node shoot segments were used as explants. Five explants were placed vertically in each vessel. A total of 25 explants per treatment were cultured in 5 vessels, resulting in 550 explants in total. The previously defined growth parameters (SN, SL, RL, NN, RN, SW, RW, CW, LN, LL, LW) were recorded throughout the experiment, which lasted for four weeks.

The optimization of the shoot propagation phase of the in vitro protocol was performed using a sequential, three-phase experimental design (mineral composition, carbohydrate source and cytokinin interactions). In each phase, the selection of optimal treatment combinations was based on the highest propagation rate (number of shoots and nodes per explant) and excellent morphological quality, with particular attention to plants with high phenotypic uniformity, low hyperhydration rate and avoiding excessive callus formation at the base of the explant.

### 2.5. In Vitro Rooting of In Vitro Shoots

Rooting was induced on half-strength MS culture medium supplemented with 2% saccharose, with or without 0.55 µM (0.1 mg L^−1^) NAA. Five propagated shoots were placed vertically in each vessel. A total of 30–40 explants were used per treatment, cultured in 6–8 vessels. The total number of explants used in this experiment was 150. Cultures were maintained under standard culture room conditions. Rooting was visually assessed on days 7 and 13. The experiment lasted for 13 days.

### 2.6. Hardening of In Vitro Rooted Plantlets

Rooted in vitro plantlets were transplanted into pots (10 cm diameter × 8 cm depth) filled with planting soil (Mr. Garden, Agro CS Hungary Ltd., Salgótarján, Hungary). They were irrigated with a starter solution containing half-strength MS macro- and microsalts and 1.5 mL of Previcur Energy (Bayer Hungária Kft. Budapest, Hungary), dissolved in 1 L of distilled water. Potted plantlets were maintained in a growth room under a 16/8 h light/dark cycle at 23 ± 2 °C. Lighting was provided by a 1:1 mixture of daylight and warm-white fluorescent tubes, resulting in a light intensity of 130 µmol m^−2^ s^−1^. Humidity was maintained by covering the pots with transparent plastic caps for the first six days. After six days, the covers were removed, and relative humidity was maintained at 65–70% using a humidifier. The experiment lasted for four weeks.

### 2.7. Statistical Analysis of Data

Data were collected as described in the above sections. For data analysis, one-way ANOVA followed by Tukey’s test at *p* < 0.05 was used to compare the effects of various treatments or treatment combinations. While the experimental design included multiple factors (e.g., cytokinin type, concentration, and carbon source), each combination was treated as an independent group. This approach was chosen to facilitate the direct comparison of all treatment protocols and to identify the optimal combination for multiplication, prioritizing the selection of the most effective treatment over the analysis of interactions between individual factors. In the latter case, each treatment combination was considered a separate group. SPSS for Windows (version 21.0) was used for statistical analysis.

## 3. Results

### 3.1. Seed Germination and Establishment of In Vitro Cultures

Seed germination was 87% after seven days on wetted filter paper, without seed disinfection. Seed germination decreased to 32.7% seven days after placement on culture medium, without any contamination, when the first method of surface disinfection was used. However, using the second disinfection method, the contamination was 52%.

### 3.2. Investigation of Minerals of the Shoot Multiplication Medium

**Figure 1:**
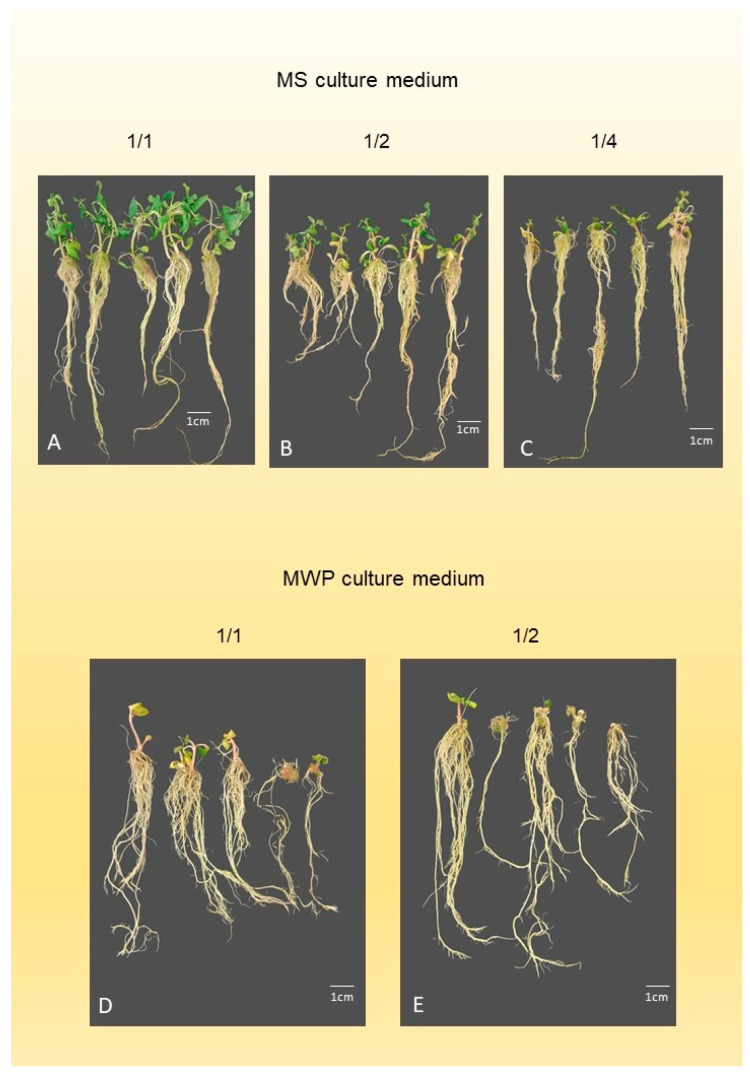
Effects of mineral composition in the shoot multiplication medium on in vitro growth. (**A**) Shoots grown on full-strength MS medium (1/1 MS). (**B**) Shoots grown on half-strength MS medium (½ MS). (**C**) Shoots grown on quarter-strength MS medium (¼ MS). (**D**) Shoots grown on full-strength MWP medium (1/1 MWP). (**E**) Shoots grown on half-strength MWP medium (½ MWP).

Shoot number (SN) of *A. oleracea* was the highest (1.5 ± 0.08) when cultured on full-strength MS medium (Figure 1A, Table 2). Half- and quarter-strength MS macro salts resulted in similar shoot numbers (1.3 ± 0.07 and 1.10 ± 0.08, respectively) (Figure 1B,C). The lowest SN (0.6 ± 0.07) was obtained on half-strength MWP medium (Figure 1E). Shoot length (SL) was the highest (70.2 ± 3.72 mm) on full-strength MS medium. Half- and quarter-strength MS media resulted in lower SL values (44.0 ± 2.69 mm and 32.5 ± 2.62 mm, respectively). MWP (Figure 1D) and half-strength MWP macro salts further reduced SL (18.7 ± 2.08 mm and 12.6 ± 1.66 mm, respectively). Node number (NN) was the highest (3.6 ± 0.19) on full-strength MS medium, while the lowest NN (0.9 ± 0.12) was observed on half-strength MWP medium. Leaf number (LN) was also highest on full-strength MS medium (7.4 ± 0.37), whereas the lowest value (1.8 ± 0.22) was observed on half-strength MWP medium. Root length (RL) was the highest (259.8 ± 27.62 mm) on full-strength MS medium. All other medium compositions resulted in lower RL values. Root number (RN) was significantly higher on all MS media.

Higher amounts of callus were produced when explants were cultured on full or half-strength MWP medium (Figure 1D,E) (45.0% and 54.4%, respectively) (Table 3) compared to all MS media types (Figure 1A–C). The hyperhydration percentage of explants (H) was highest on full-strength MWP medium (20.0%), whereas all other media resulted in lower values. The bud break percentage (B) was highest on half-strength MS (88.0%) and full-strength MS (87.0%) media (Figure 1A,B). A lower B value (78.0%) was recorded on quarter-strength MS medium (Figure 1C). Shoot regeneration (45.0%) was the lowest on MWP medium (Figure 1D). The callus production among explants regenerating shoots (CS) was the lowest (0%) on half-strength MS medium (Figure 1B). The percentage of hyperhydrated shoots among regenerated shoots (HHS) was highest (3.0%) on full-strength MWP medium (Figure 1D).

### 3.3. Investigation of the Carbon Source of the Shoot Multiplication Medium

**Figure 2:**
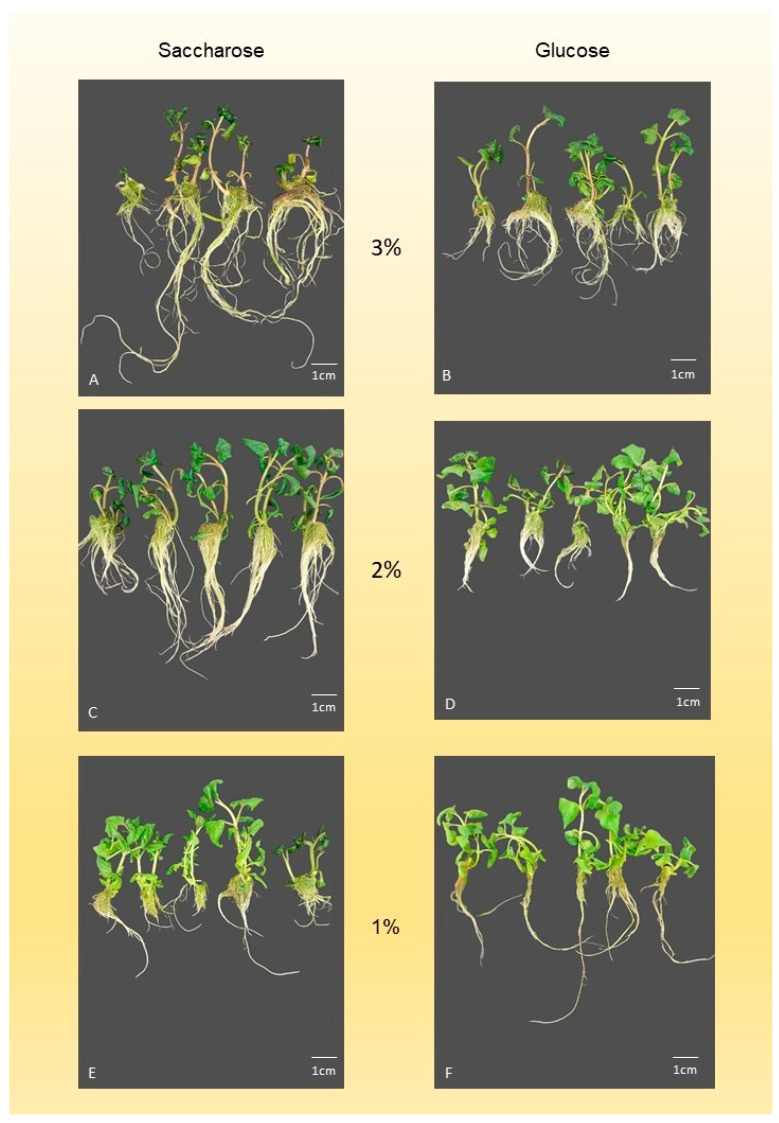
Effects of the carbon source of the multiplication medium on shoot growth and multiplication. (**A**) Shoots grown on medium containing 3% saccharose. (**B**) Shoots grown on medium containing 3% glucose. (**C**) Shoots grown on medium containing 2% saccharose. (**D**) Shoots grown on medium containing 2% glucose. (**E**) Shoots grown on medium containing 1% saccharose. (**F**) Shoots grown on medium containing 1% glucose.

The highest shoot number (SN) (1.8 ± 0.07) was obtained on medium containing 2% of saccharose (Figure 2C) (Table 4), but a similar result was observed with 1% saccharose (Figure 2E) or 1% glucose (Figure 2F). The lowest SN (1.2 ± 0.09) was obtained on medium containing 3% of glucose (Figure 2B). The highest shoot length (SL) (62.6 ± 1.66 mm) was recorded on medium containing 2% glucose. A comparable SL was observed with 3% saccharose (Figure 2A). The lowest SL (46.7 ± 2.54 mm) was measured on 3% glucose (Figure 2B), while 2% saccharose (Figure 2C) and 1% glucose (Figure 2E) produced similar values. The highest node number (NN) was obtained on media containing 2% glucose (Figure 2D) (3.61 ± 0.06), 1% glucose (Figure 2F) (3.6 ± 0.08), or 1% saccharose (Figure 2E) (3.57 ± 0.08). The lowest NN (2.6 ± 0.12) was recorded on medium containing 3% glucose (Figure 2B). The highest leaf number (LN) was found on medium supplemented with 2% glucose (Figure 2D) (7.2 ± 0.12), 1% glucose (Figure 2F) (7.1 ± 0.16), or 1% saccharose (Figure 2E) (7.2 ± 0.16). The lowest LN (5.4 ± 0.24) occurred on medium with 3% glucose (Figure 2B). Total shoot number (TSN) was highest on 2% (Figure 2C) (8.9 ± 0.32) and 1% saccharose (Figure 2E) (8.2 ± 0.46), with no significant difference between them. The lowest TSN (6.1 ± 0.44) was found on 3% glucose (Figure 2B). Root length (RL) was the longest at 3% saccharose. (Figure 2A) (228.3 ± 9.43 mm), but 2% saccharose also resulted in similar RL values. The lowest RL was observed on medium with 3% glucose (Figure 2B) (66.6 ± 3.89 mm) and 1% saccharose (Figure 2E) (60.9 ± 2.63 mm). The highest root number (RN) was recorded on 3% saccharose (Figure 2A) (28.1 ± 1.57), but 2% and 1% saccharose produced comparable results. The lowest RN (17.2 ± 1.24) was obtained on 3% glucose (Figure 2B). Shoot weight (SW) reached its maximum on 3% saccharose (Figure 2A) (17,247 ± 889 mg), while the lowest SW (7170 ± 287 mg) was measured with 1% saccharose (Figure 2E). Root weight (RW) was highest on 3% saccharose (Figure 2A) (16,595 ± 1630 mg) and 2% saccharose (Figure 2C) (14,376 ± 3569 mg). All other sugar concentrations resulted in lower RW values.

Callus formation was observed at the wounding sites of the shoot explants only in the case of higher sugar concentrations (2% and 3%) (Figure 2A–D, Table 5). A callus was formed by 12.0% of explants cultured on medium containing 2% saccharose (Figure 2C), whereas only 1.1% of explants produced callus on medium with 2% glucose (Figure 2D). In the presence of 3% saccharose (Figure 2A), 25.5% of explants formed callus, and a comparable value (26.6%) was observed on medium containing 3% glucose (Figure 2B).

### 3.4. Effects of Various Cytokinins Applied in the Shoot Multiplication Medium

**Figure 3:**
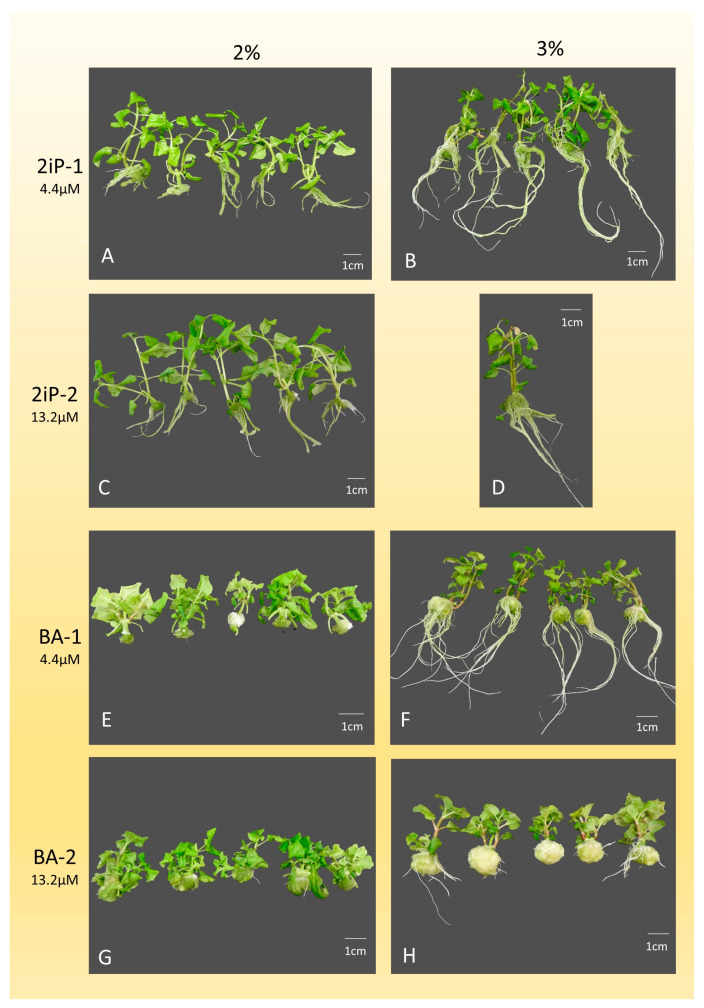
Effects of various cytokinins applied to shoot multiplication medium on shoot growth and multiplication. (**A**) Shoots grown on medium containing 2iP-1 and 2% glucose. (**B**) Shoots grown on medium containing 2iP-1 and 3% saccharose. (**C**) Shoots grown on medium containing 2iP-2 and 2% glucose. (**D**) Shoots grown on medium containing 2iP-2 and 3% saccharose. (**E**) Shoots grown on medium containing BA-1 and 2% glucose. (**F**) Shoots grown on medium containing BA-1 and 3% saccharose. (**G**) Shoots grown on medium containing BA-2 and 2% glucose. (**H**) Shoots grown on medium containing BA-2 and 3% saccharose.

**Figure 4:**
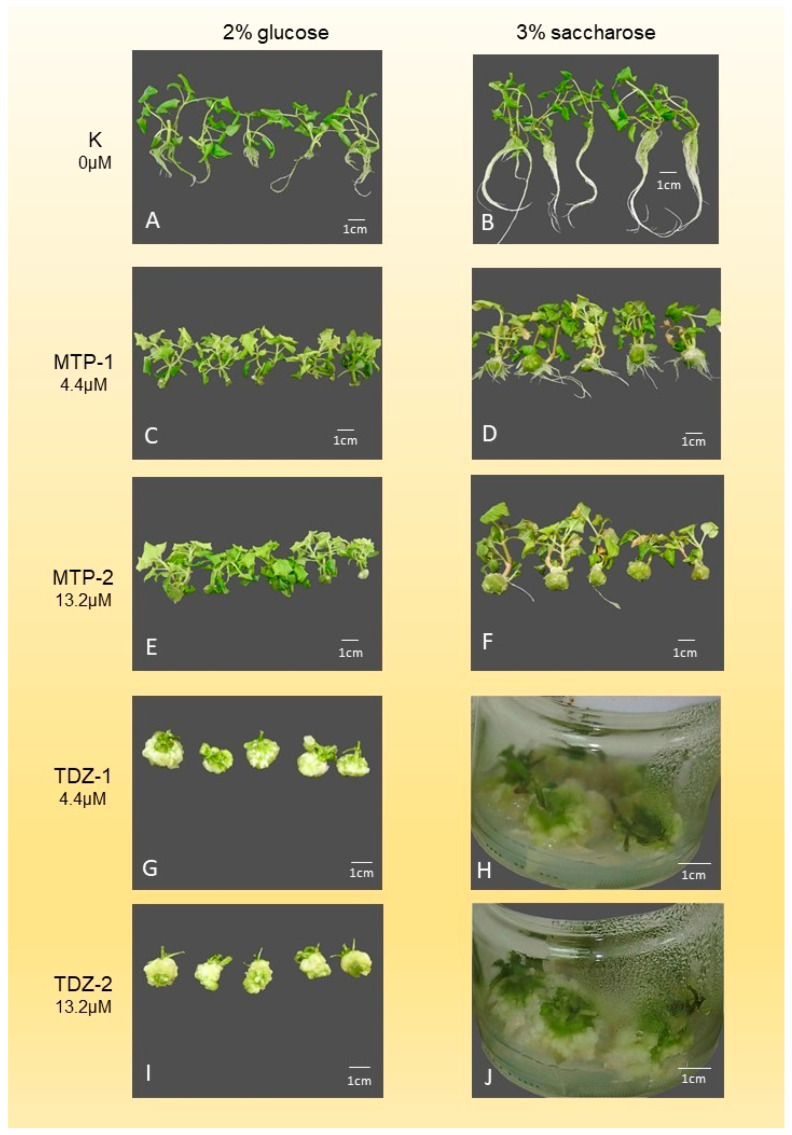
Effects of various cytokinins applied to shoot multiplication medium. (**A**) Shoots grown on K (control) medium containing 2% glucose. (**B**) Shoots grown on K (control) medium containing 3% saccharose. (**C**) Shoots grown on medium containing MTP-1 and 2% glucose. (**D**) Shoots grown on medium containing MTP-1 and 3% saccharose. (**E**) Shoots grown on medium containing MTP-2 and 2% glucose. (**F**) Shoots grown on medium containing MTP-2 and 3% saccharose. (**G**) Shoots grown on medium containing TDZ-1 and 2% glucose. (**H**) Shoots grown on medium containing TDZ-1 and 3% saccharose. (**I**) Shoots grown on medium containing TDZ-2 and 2% glucose. (**J**) Shoots grown on medium containing TDZ-2 and 3% saccharose.

**Figure 5:**
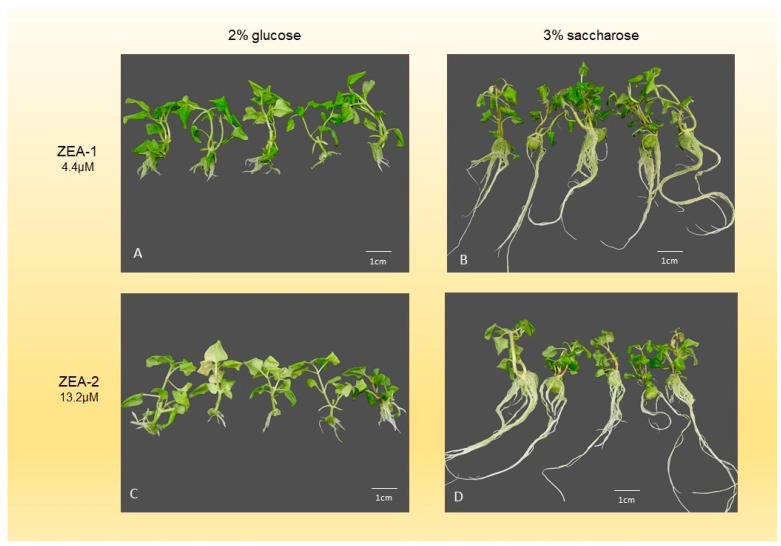
Effects of various cytokinins applied in the shoot multiplication medium on the in vitro growth. (**A**) Shoots grown on medium containing ZEA-1 and 2% glucose. (**B**) Shoots grown on medium containing ZEA-1 and 3% saccharose. (**C**) Shoots grown on medium containing ZEA-2 and 2% glucose. (**D**) Shoots grown on medium containing ZEA-2 and 3% saccharose.

The interaction of various cytokinins and carbon sources significantly influenced the morphogenetic response of *A. oleracea* (Figure 3, Figure 4 and Figure 5). When 2% glucose was used (Table 6), the highest shoot number (SN) was achieved with MTP-2 (2.5 ± 0.9), while the lowest was recorded with 2iP-1 (1.6 ± 0.6). Notably, TDZ treatments failed to produce shoots, resulting in complete explant callusing. The maximum shoot length (SL) was observed with 2iP-2 (65.6 ± 10.7 mm), whereas the control and BA treatments yielded significantly lower values. Optimal node numbers (NN) were obtained using BA-1 (9.3 ± 3.0) and BA-2 (9.7 ± 3.5). Regarding root development, 2iP-1 supported the highest root number (11.8 ± 5.0), while TDZ, MTP, and BA-2 treatments completely inhibited rooting.

In the presence of 3% sucrose (Table 7), MTP-2 again proved superior for shoot multiplication, yielding the highest SN (2.9 ± 0.9), whereas TDZ-2 resulted in the lowest (1.0). Shoot length peaked with 2iP-1 (94.6 ± 17.2 mm), significantly outperforming the TDZ treatments. The highest leaf number (LN) and leaf length (LL) were recorded with MTP-1 (20.5 ± 5.6) and MTP-2 (34.1 ± 6.5 mm), respectively. In terms of underground development, the control (K) and ZEA-1 treatments provided the most robust root systems, while MTP and BA-2 significantly suppressed root growth and weight. Similar to the glucose treatments, TDZ induced excessive callus formation (up to 6284 mg) at the expense of organogenesis.

### 3.5. In Vitro Rooting of In Vitro Shoots

**Figure 6:**
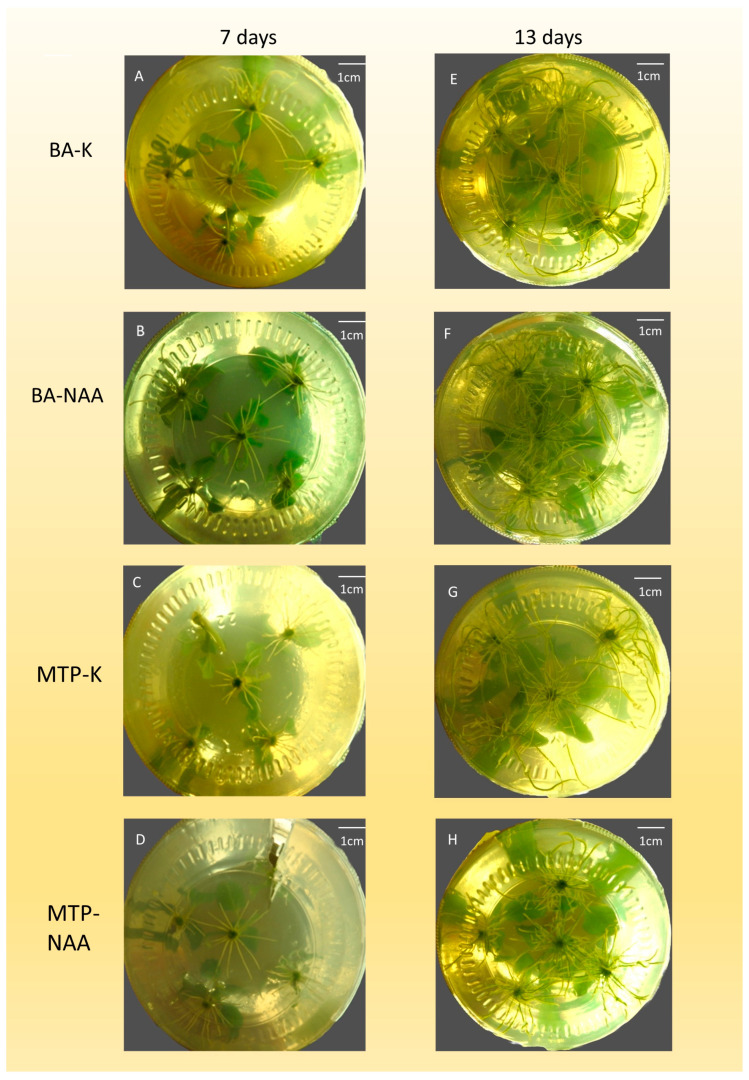
In vitro rooting of in vitro shoots. Plantlets multiplied on BA-containing medium and rooted on K (control) medium, 7 days after transfer ((**A**), BA–K) and 13 days after transfer ((**E**), BA-K). Plantlets multiplied on BA-containing medium and rooted on NAA-containing medium, 7 days after transfer ((**B**), BA–NAA) and 13 days after transfer ((**F**), BA-NAA). Plantlets multiplied on MTP-containing medium and rooted on K (control) medium, 7 days after transfer ((**C**), MTP–K) and 13 days after transfer ((**G**), MTP-K). Plantlets multiplied on MTP-containing medium and rooted on NAA-containing medium, 7 days after transfer ((**D**), MTP–NAA) and 13 days after ((**H**), MTP-NAA).

All shoots multiplied on media containing BA began to root after 5 days when NAA was added to the rooting medium (Figure 6B,F). Most shoots propagated on BA-containing media also initiated rooting after 5 days on the control medium without NAA, but rooting was uneven (Figure 6A,E). Shoots, multiplied on MTP-containing media, developed roots at higher rates and higher length when NAA was present in the rooting medium (Figure 6D,H); however, root formation also occurred without NAA, but at a lower rate and shorter root length (Figure 6C,G). The shoot number (SN) of plantlets propagated on BA- or MTP-containing media showed no significant difference between treatments with or without NAA in the rooting medium (Table 8, Figure 6A–H). However, shoot length (SL) was influenced both by the residual effect of the cytokinins used in the multiplication medium and the presence or absence of NAA in the rooting phase.

The shoot length (SL) was the highest (34.0 ± 1.5 mm) in the case of plantlets propagated on BA-containing medium and rooted on medium without NAA (Figure 6A,E). The lowest SL (29.2 ± 1.7 mm) was observed when plantlets were multiplied on MTP-containing medium and rooted on medium lacking NAA (Figure 6C,G).

The node number (NN) was higher (BA: 4.2 ± 0.29, MTP: 4.0 ± 0.15) on culture medium, containing NAA, regardless of the type of PGR used in the multiplication phase (Figure 6B,D,F,H). Plantlets propagated on BA-containing media produced the highest shoot weight (SW) (562 ± 65 mg) when NAA was included in the rooting medium (Figure 6B,F). The type of cytokinins applied in the multiplication medium or the presence or absence of NAA applied in the rooting medium did not affect the root number (RN) of plantlets. However, the root length (RL) of the plantlets was higher (BA: 33.4 ± 1.0 mm, MTP: 35.9 ± 1.7 mm) when NAA was absent from the rooting medium (Figure 6A,C,E,G). The root weight (RW) of rooted plantlets was higher (BA: 202 ± 23 mg, MTP: 193 ± 14 mg) in the presence of NAA in the rooting medium (Figure 6B,D,F,H). In contrast, the lowest RW (90 ± 6 mg) was recorded when plantlets propagated on BA-containing media were rooted without NAA (Figure 6A,E). The leaf number (LN) of rooted plantlets propagated on BA-containing media was the highest (10.6 ± 1.4) when NAA was present in the rooting medium (Figure 6B,F). Other combinations of cytokinins and NAA did not significantly influence LN. The leaf length (LL) of rooted plantlets did not differ significantly between treatments. The leaf width (LW) was higher (BA: 15.7 ± 0.8 mm, MTP: 15.5 ± 0.5 mm) when NAA was present in the rooting medium (Figure 6B,D,F,H). The lowest LW (12.7 ± 0.8 mm) was observed in plantlets multiplied on MTP-containing media and rooted without NAA (Figure 6C,G).

### 3.6. Hardening of In Vitro Rooted Plantlets

**Figure 7:**
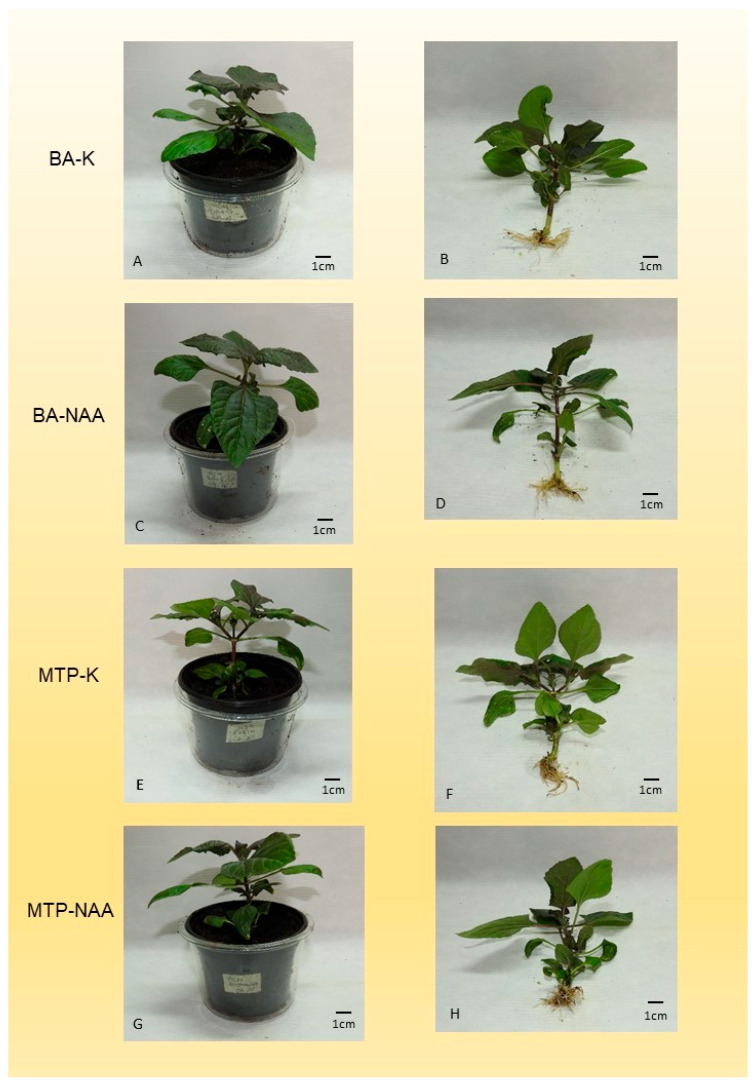
Hardened, in vitro multiplied and rooted plants after 4 weeks. ((**A**,**B**), BA-K) Plant, propagated on medium, containing BA, then rooted on K (control) medium. ((**C**,**D**), BA-NAA) Plant, propagated on medium, containing BA, then rooted on medium, containing NAA ((**E**,**F**), MTP-K). Plant, propagated on medium, containing MTP, then rooted on K (control) medium. ((**G**,**H**), MTP-NAA) Plantlets propagated on medium, containing MTP, then rooted on medium, contained NAA.

Plants were slightly droopy when plastic covers were removed 6 days after planting, but they straightened up after 2 days. Branching was the highest in the case of hardened plants multiplied on medium supplemented with BA and then rooted in the presence of NAA (Figure 7C,D) (Table 9). The shoot length of plants was higher when NAA was not present in the rooting medium, regardless of the type of cytokinin used in the multiplication medium (Figure 7A,B,E,F). The number of hardened plants did not differ after treatments. The shoot weight (SW) was the highest when NAA was not applied in the rooting medium, regardless of the type of cytokinin used in the multiplication medium (Figure 7A,B,E,F). The tendency of the hardened plants to form flower buds was the same in all treatments. The leaf length (LL) and leaf width (LW) of the second pair leaf, the root length (RL), or the root weight (RW) did not differ after treatments. The potted plants started to form flower buds 1 month after planting, and the flowers opened fully after 3 months.

## 4. Discussion

### 4.1. Seed Germination and Establishment of In Vitro Cultures

The natural germination capability of *A. oleracea* seeds was quite high (87%), but it decreased considerably (32%) after the first disinfection method, using HgCl_2_ in 0.1% concentration. This may have been due to the high toxicity of HgCl_2_, but it had the advantage that no contamination could be detected when using it. Sharma et al. [27] applied a similar method. However, contamination was detected after the second method using household sodium hypochlorite in 1/3 strength; hence, the first method proved to be better for us to obtain plantlets free of contamination. Joshi et al. [29] obtained similar results. It is important to highlight that mercuric chloride is a highly toxic and hazardous substance that can kill most living organisms. It should be handled with care, and only the amount strictly necessary should be used. However, its use is advantageous during surface sterilization because it is extremely effective against contaminants. The HgCl_2_ solution should be handled after use in accordance with the instructions for the storage and disposal of hazardous substances. For laboratories where mercury use is restricted, sodium hypochlorite serves as a viable, mercury-free alternative. Although our preliminary tests indicated that NaOCl might result in lower survival rates (with contamination levels reaching 52%), its application significantly reduces chemical hazards while providing an acceptable level of decontamination if exposure times are further optimized.

### 4.2. Investigation of Minerals of the Shoot Multiplication Medium

*A. oleracea* plantlets grew vigorously on PGR-free, full-strength MS medium supplemented with 3% saccharose, without significant malformation or callus production, and spontaneously rooted well. A similar result was observed in the case of *A. radicans* var. *radicans* by Rios-Chavez et al. [30]. All measured growth parameters (Table 2) were the highest, most of them significantly, on full-strength MS medium with 3% saccharose content, compared to half- and quarter-strength MS or full- and half-strength MWP media. The values of the measured shoot growth parameters (shoot number, shoot length, node number, leaf number) decreased gradually from the full-strength MS medium to the half-strength MWP medium. MS medium contains a high amount of inorganic nitrogen, partly in the form of potassium nitrate (KNO_3_, 1900 mg L^−1^) and ammonium nitrate (NH_4_NO_3_, 1650 mg L^−1^), with a total nitrate content of 3550 mg L^−1^. In contrast, the MWP medium contains a lower amount of total inorganic nitrogen (871 mg L^−1^) in a different composition: 471 mg L^−1^ calcium nitrate (Ca(NO_3_)_2_) and 400 mg L^−1^ ammonium nitrate (NH_4_NO_3_). This means it contains only about a quarter of the total nitrogen compared to MS medium. Our results (Table 2) may suggest that nitrogen content strongly influenced the shoot growth parameters, especially shoot elongation, as described by Pasternak et al. [35]. The high shoot regeneration percentage of explants (Table 3) cultured on half-strength and full-strength MS culture medium also confirmed that a higher nitrogen level enhances shoot regeneration. Additionally, MWP medium contains a higher sulphate content, mainly in the form of potassium sulphate (K_2_SO_4_). Another critical factor is the total salt concentration of the culture medium, which affects overall shoot quality and root development. MS culture medium contains 980 mg L^−1^ of non-nitrate salts, resulting in a total salt content of 4530 mg L^−1^, classifying it as a high-salt medium. In contrast, MWP medium contains 1412.5 mg L^−1^ of non-nitrate salts, and its total salt content is 2283.5 mg L^−1^—roughly half that of MS medium. Higher salt concentrations may induce callus formation or hyperhydricity [35]. Our findings showed that callus formation was lowest (11%) on half-strength MS medium. Full-strength MS medium did not cause a significant increase in callus formation either (13%) (Table 3). In contrast, the MWP medium caused substantially more callus development (45–54.4%) (Table 3), indicating that MS basal salt composition is more suitable for *A. oleracea* culture. The lowest percentage of hyperhydrated explants was observed on quarter-strength MS medium (5.3%), but a similarly low percentage (8.0%) was recorded on full-strength MS medium. These results confirm that lower total salt content promotes root growth [30]. Therefore, half-strength MS or full-strength MWP medium is recommended for rooting. Additionally, culturing on half-strength MS medium may be beneficial to prolong the culture period when the goal is maintenance rather than rapid multiplication.

### 4.3. Investigation of the Carbon Source of Shoot Multiplication

Carbohydrates help in the regeneration of plant parts. Small plant parts, which contain only a small amount of chlorophyll, or those that do not have photosynthetic activity, like dividing tissues, need to have carbohydrates added to the plant culture medium for growth [36]. Carbohydrates may cause malformed or hyperhydrated tissues or may cause uneven shoot structure [15]. Our results showed that medium or low amounts of carbohydrates resulted in better shoot growth of *A. oleracea* from one-node segments without growth regulators, but higher amounts reduced it (Table 4). The node number was higher on 1% saccharose, or 1–2% glucose. This may be the result of shorter internodes and a more compact shoot structure caused by the lower energy supply (Figure 2). The leaf number was also the highest at low carbohydrate content (2% or 1% of glucose or 1% of saccharose). The higher node number may have allowed for the development of a higher leaf number. Saccharose applied at 1% or 2% in the culture medium caused higher callus formation and higher hyperhydration than glucose; however, almost the same results were observed at 3% saccharose and glucose (Table 5). In addition to shoot number and node number, biomass and shoot length are also important parameters for the final shoot yield. Furthermore, for shoot quality and genetic fidelity, it is important to keep callus formation and hyperhydration rates low (below 30%). Basal callus formation was considered undesirable in this context, as the protocol aims to promote direct organogenesis in order to support genetic fidelity. Extensive callusing at the explant base may interfere with nutrient allocation and affect the overall quality of regenerated shoots; therefore, treatments minimizing callus formation while maximizing shoot multiplication were regarded as optimal. *A. oleracea* could grow in the presence of glucose, but at higher concentrations, it resulted in lower growth parameters compared to saccharose. Thus, we applied 3% saccharose or 2% glucose for shoot multiplication in further experiments to investigate the effect of cytokinins. A similar growth characteristic was obtained in the experiment conducted by Mohan et al. [15] in the case of *Acmella ciliata*. Shoot development decreased with increasing saccharose concentration. Additionally, they found that 3% saccharose content in multiplication medium is the optimum sugar content, since they measured the highest shoot multiplication rate on medium supplemented with 3% saccharose.

### 4.4. Effects of Various Cytokinins Applied in the Shoot Multiplication Medium

The most important parameters in shoot multiplication, which determine the multiplication rate, are the shoot number (SN) and the node number (NN). The highest average shoot number (2.9 ± 0.88) with a 100% response rate was obtained on a medium containing MTP at a higher concentration (MTP-2: 13.2 µM) with 3% saccharose, within 4 weeks. This treatment also produced an average shoot length (SL) of 41.3 ± 10.0 mm and 7.2 ± 2.95 nodes per shoot. When MTP was applied at a lower concentration (MTP-1: 4.4 µM), the average shoot number was slightly lower (2.7 ± 1.07), but it resulted in longer shoots (49.0 ± 17.0 mm) and a higher node number (8.0 ± 2.24). The explant response rate remained 100% in this case as well. These results suggest that increasing the concentration of MTP can increase shoot number but may reduce node number—ultimately yielding a similar multiplication rate. Therefore, further increases in MTP concentration are not recommended.

The highest node number (9.7 ± 3.51) was recorded on medium containing a higher concentration of BA (BA-2: 13.2 µM) with 2% glucose. In this treatment, the shoot number (2.4 ± 0.81) and shoot length (32.0 ± 7.3 mm) were slightly lower, but not significantly different from the values achieved with MTP. The explant response was also 100% in the BA treatment. Thus, BA with 2% glucose is recommended for shoot multiplication of *A. oleracea*. However, increasing the BA concentration may further affect shoot and node numbers.

Comparative studies further support our findings. For instance, Algabri et al. [24] reported 4.3 ± 0.08 shoots with a 35% explant response rate using 1 mg L^−1^ BA, while a higher concentration (3 mg L^−1^) increased the response rate to 90% but reduced the shoot number to 2.0. Similarly, Kumar et al. [15] achieved 4.42 ± 0.4 shoots in *A. ciliata* with 0.5 mg L^−1^ BA, which is comparable to the 7.12 shoots obtained by Razaq et al. [32] using 10 µM BA over a 6-week period. Furthermore, Nabi et al. [26] reported 6.9 ± 3.2 shoots on MS medium supplemented with 0.5 mg L^−1^ BA and 0.5 mg L^−1^ IBA. However, a common trend in several studies [26,29] is that increasing BA concentrations beyond an optimal level leads to a decline in shoot and leaf numbers. For example, Joshi et al. [29] observed a significant drop in leaf count from 10.31 to 6.41 when BA was increased from 1 to 2 mg L^−1^, a phenomenon that aligns with our own observations regarding hormonal inhibition.

In our experiments, 17.9 ± 3.6 leaves were obtained at 4.4 µM BA (1 mg L^−1^), when culture media were complemented with 3% saccharose, while this number dropped to 11.8 ± 2.5 at 13.2 µM BA (3 mg L^−1^).

TDZ led to high callus production at both tested concentrations. Sharma and Shahzad [27] observed a similar trend—strong callus production and adventitious shoots at lower concentrations of TDZ. Therefore, TDZ may be suitable for shoot multiplication at lower concentrations, but its strong callus-inducing effect may compromise genetic fidelity and increase the risk of somaclonal variation.

ZEA and 2iP resulted in equal or fewer shoots than the control, suggesting they may not be suitable for *A. oleracea* propagation. Similar results with 2iP were reported by Razaq et al. [32].

### 4.5. In Vitro Rooting of In Vitro Shoots

Although rooted shoots were also formed on PGR-free media or even on multiplication media (in the case of ZEA or 2iP), the development of healthy roots requires the addition of auxin to the rooting medium [27,29]. In our experiment, ½-strength MS medium supplemented with 0.55 µM (0.1 mg L^−1^) NAA was used for rooting. A similar rooting medium was applied by Sharma et al. [27]. According to Nabi et al. [21], full-strength MS medium was more effective for root induction than ½-strength MS, as they achieved 70% rooting on MS medium supplemented with 0.5 mg L^−1^ IBA. Sharma et al. [27] compared the effects of NAA, IBA, and IAA on rooting and found that the highest number and length of roots were achieved on ½ MS medium supplemented with 2.5 µM NAA. Additionally, plants rooted on NAA-containing media exhibited the highest survival rate (96%).

The putative after-effects of cytokinins were observed in some growth parameters of rooting in our experiment (Table 7). The shoot length of plantlets was the highest when shoots were multiplied on media containing BA or MTP, and NAA was not applied in the rooting medium. The application of NAA equalized the differences in root length between plantlets multiplied on media containing BA or MTP. A similar tendency was observed by Joshi et al. [29]. The node number was influenced by NAA since plantlets had a higher node number when NAA was present in the rooting medium. NAA also influenced the shoot weight, but some putative after-effects of MTP were observed too. Plantlets multiplied on media containing MTP reached lower shoot weight when NAA was applied in the rooting medium compared to plantlets multiplied on media containing BA.

The length of roots was influenced mainly by NAA. All of the plantlets rooted on the NAA-containing medium had smaller root length, compared to plantlets rooted without NAA. Sharma et al. [27] obtained reverse results, because they measured lower root number and root length when NAA was not applied in the rooting medium. It may be caused by the after-effect of cytokinins used in the multiplication medium. In the case of root mass, a strong cytokinin after-effect can be assumed. Plantlets multiplied on media containing BA resulted in about half of the root weight compared to plantlets multiplied on media containing MTP. Still, the application of NAA in the rooting medium partly equalized the differences between them. The after-effect of BA was presumably in the leaf numbers, because only plantlets multiplied on media containing BA developed significantly elevated leaf numbers when NAA was applied in rooting media. Leaf width may also have been influenced by cytokinins used in the propagation medium, because plantlets multiplied on BA-containing media had higher leaf width when NAA was not applied in rooting media, compared to plantlets multiplied on MTP-containing media. To counteract the likely after-effects of cytokinins, the use of NAA in the rooting medium is recommended.

### 4.6. Hardening of In Vitro Rooted Plantlets

The after-effect of cytokinins was also likely in the growth parameters of acclimatized plants. Increased branching was observed only in the case of plants multiplied on BA-containing media and then rooted on medium that contained NAA. The shoot length may have been influenced by NAA, because significantly higher shoot length was measured in plants rooted on medium that contained NAA. The presumable after-effect of BA was observed relating to the shoot weight, as well. The shoot weight was significantly higher only when shoots were multiplied on media containing BA and rooted on media containing NAA. A small but non-significant effect of NAA could be observed in the number of flower buds, in the length and width of leaves, and in the length of roots. The differences in the growth parameters of plants were partly equalized under the process of hardening. The application of NAA can be proposed in the rooting, because it helps reduce the likely after-effects of cytokinins applied during shoot multiplication.

**Figure 8:**
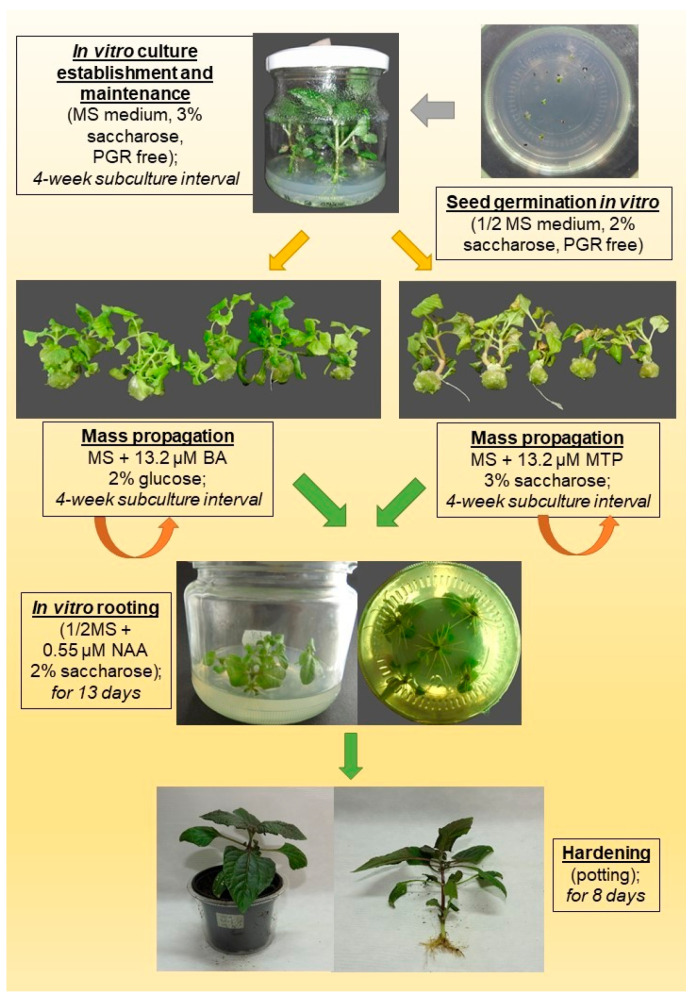
Schematic representation of the optimized in vitro propagation protocols for *Acmella oleracea* (L.) R.K. Jansen including the duration and specific conditions for each stage of micropropagation. Red arrows indicate two alternative pathways for mass shoot multiplication; green arrows indicate the subsequent steps for rooting and acclimatization of developed plantlets.

In this study, an efficient in vitro micropropagation protocol (Figure 8) was developed for *Acmella oleracea* (L.) R.K.Jansen, in which two alternatives are proposed for shoot multiplication. Surface-sterilized seeds (0.1% HgCl_2_ for 4 min, followed by 70% ethanol for 30 s) should be germinated on half-strength MS culture medium, enriched with 2% saccharose. Nodal shoot segments from seedlings can be used as explants in vitro. Shoot multiplication was the most efficient at applications of 13.2 µM meta-Topolin with 3% saccharose or 13.2 µM Benzyladenine with 2% glucose in full-strength MS medium. In vitro multiplied shoots are proposed to root in vitro on half-strength MS medium containing 0.55 µM NAA within 13 days. Rooted shoots can be 100% acclimatized within 8 days.

## 5. Conclusions

In response to the growing demand for natural and effective bioactive ingredients in the cosmetic industry, we have developed an efficient in vitro propagation protocol for *A. oleracea*. Protocol development was based on a multi-parameter evaluation approach, considering multiplication rate per 4-week culture cycle, explant response percentage, morphological stability (limited basal callusing and hyperhydricity), and acclimatization success, rather than shoot number alone. The effects of basal media (MS, MWP, and their semi-concentrated versions), carbohydrate sources (saccharose and glucose at 1–3%), and different cytokinin types (BA, TDZ, ZEA, TOP and 2iP) on shoot growth and multiplication, and their subsequent effects on rooting, as well as rooting treatments with or without NAA, were investigated. Two in vitro protocols may be proposed for the micropropagation of *Acmella oleracea* (L.) R.K.Jansen.

The developed protocol (Figure 8) offers a superior multiplication rate compared to previously reported methods for *A. oleracea*. The method achieved a 100% axillary shoot regeneration frequency from the explants, yielding 2.9 healthy shoots per nodal explant with a high number of nodes per shoot (up to 8) within a short, 4-week-long cycle. This high nodal productivity implies that a single shoot can potentially yield more than 20 new plantlets in each subsequent multiplication subculture. This ensures a significant pool of potential explants while maintaining high plantlet quality, phenotypic uniformity, and 100% acclimatization success. This optimized method facilitates future industrial applications, providing a contamination-free and standardized source of biomass as a reliable foundation for future phytochemical validation in the cosmetic and pharmaceutical industries.

## Figures and Tables

**Table 1:** Steps of surface disinfection procedures.

Step	Procedure 1.	Procedure 2.
1.	Ultra Sol™, for 5 min.	
2.	0.1% HgCl_2_, for 4 min.	1/3 NaOCl, for 6 min.
3.	SDW, for 1 min.	
4.	70% ethanol, for 30 s	
5.	SDW, 3 × for 10 min. each	

**Table 2:** Mean values of measured parameters with standard errors in the experiment of mineral composition of culture media. Different letters following the data indicate statistically significant differences (*p* < 0.05) between treatments according to ANOVA and Tukey tests. (Abbreviations: SN: shoot number, SL: shoot length, NN: node number, LN: leaf number, RL: root length, RN: root number).

Mineral Composition	SN (pcs/Explant)	SL (mm/Shoot)	NN (pcs/Shoot)	LN (pcs/Shoot)	RL (mm/Explant)	RN (pcs/Explant)
1. 1/2MWP	0.6 ± 0.07 c	12.6 ± 1.66 d	0.9 ± 0.12 d	1.8 ± 0.22 d	156.8 ± 7.53 b	15.1 ± 0.93 b
2. MWP	0.8 ± 0.07 c	18.7 ± 2.08 d	1.4 ± 0.14 d	2.7 ± 0.26 d	154.8 ± 8.95 b	12.2 ± 0.67 b
3. 1/4MS	1.1 ± 0.08 b	32.5 ± 2.62 c	2.1 ± 0.15 c	4.5 ± 0.29 c	201.4 ± 9.14 b	20.2 ± 1.37 a
4. 1/2MS	1.3 ± 0.07 a,b	44.0 ± 2.69 b	2.9 ± 0.15 b	5.9 ± 0.28 b	197.9 ± 8.07 b	19.2 ± 0.84 a
5. MS	1.5 ± 0.08 a	70.2 ± 3.72 a	3.6 ± 0.19 a	7.4 ± 0.37 a	259.8 ± 27.62 a	22.1 ± 1.08 a

**Table 3:** The percentage of explants producing callus, shoots, or both callus and shoots, as well as the percentage of hyperhydrated shoots. (Abbreviations: C: percentage of explants producing callus; H: percentage of explants showing hyperhydration symptoms; B: percentage of explants regenerating shoots; CS: percentage of explants producing both callus and shoots; HHS: percentage of regenerated shoots showing hyperhydration symptoms).

Type of Culture Media	C (%)	H (%)	B (%)	CS (%)	HHS (%)
1. 1/2MWP	54.4	10.0	47.7	3.3	0
2. MWP	45.0	20.0	45.0	4.0	3.0
3. 1/4MS	15.8	5.3	78.9	2.1	0
4. 1/2MS	11.0	11.0	88.0	0	0
5. MS	13.0	8.0	87.0	5.0	0

**Table 4:** Different letters following the data indicate statistically significant differences (*p* < 0.05) between treatments, according to ANOVA and Tukey tests. (Abbreviations: sac: saccharose; SN: shoot number; SL: shoot length; NN: node number; LN: leaf number; TSN: total shoot number; RL: root length; RN: root number; SW: shoot weight; RW: root weight.).

Sugar Content		SN (pcs/Explant)	SL (mm/Shoot)	NN (pcs/Shoot)	LN (pcs/Shoot)	TSN (pcs/Vessel)	RL (mm/Explant)	RN (pcs/Explant)	SW (mg/Vessel)	RW (mg/Vessel)
saccharose	1%	1.6 ± 0.06 a,b	54.8 ± 1.56 b	3.6 ± 0.08 a	7.2 ± 0.16 a	8.2 ± 0.46 a	60.9 ± 2.63 d	22.5 ± 0.67 b	7170 ± 287 d	3477 ± 186 b
	2%	1.8 ± 0.07 a	54.1 ± 1.68 b,c	3.0 ± 0.07 b	6.2 ± 0.13 b	8.9 ± 0.32 a	142.8 ± 4.75 b	22.9 ± 0.99 b	12,337 ± 434 b	14,376 ± 3569 a
	3%	1.3 ± 0.09 c,d	55.8 ± 2.84 a,b	3.2 ± 0.14 b	6.3 ± 0.27 b	6.4 ± 0.58 b	228.3 ± 9.43 a	28.1 ± 1.57 a	17,247 ± 889 a	16,595 ± 1630 a
glucose	1%	1.5 ± 0.06 b,c	53.1 ± 1.77 b,c	3.6 ± 0.08 a	7.1 ± 0.16 a	7.5 ± 0.30 a,b	110.9 ± 3.19 c	18.1 ± 0.67 c,d	7894 ± 125 c,d	3008 ± 77 b
	2%	1.3 ± 0.06 c,d	62.6 ± 1.66 a	3.6 ± 0.06 a	7.2 ± 0.12 a	6.4 ± 0.23 b	110.4 ± 3.74 c	21.4 ± 0.92 b,c	8573 ± 258 c,d	5165 ± 267 b
	3%	1.2 ± 0.09 d	46.7 ± 2.54 c	2.6 ± 0.12 c	5.4 ± 0.24 c	6.1 ± 0.44 b	66.6 ± 3.89 d	17.2 ± 1.24 d	9363 ± 365 c	6782 ± 497 b

**Table 5:** The percentage of explants producing callus or showing hyperhydrated symptoms on culture media containing 1, 2, or 3% of saccharose or glucose.

Sugar Content and Concentration	Saccharose		Glucose	
	Callus (%/Explant)	Hyperhydration (%/Shoot)	Callus (%/Explant)	Hyperhydration (%/Shoot)
1%	0	5.5	0	0
2%	12	7	1.1	1.1
3%	25.5	20	26.6	13

**Table 6:** Mean values of measured parameters with standard errors in the effects of various cytokinins applied in the shoot multiplication medium on shoot growth and multiplication experiment with 2% glucose content. Different letters following the data indicate significantly (*p* < 0.05) different values between treatments according to ANOVA and Tukey tests. (Abbreviations: SN: shoot number, SL: shoot length, NN: nodal number, SW: shoot weight, RN: root number, RL: root length, RW: root weight, CW: callus weight, LN: leaf number, LL: leaf length, LW: leaf width, ZEA: Zeatin, BA: Benzyladenine, TDZ: Thidiazuron, MTP: meta-Topolin, 2iP: N6-(2-Isopentenyl)adenine, K: control, cytokinins-1: 4.4 µM concentration, ytokinins-2: 13.2 µM concentration).

Cytokinin Content of the Media	SN (pcs/Explant)	SL (mm/Shoot	NN (pcs/Shoot)	SW (mg/Shoot)	RN (pcs/Explant)	RL (pcs/Explant)	RW (mg/Explant)	CW (mg/Explant)	LN (pcs/Shoot)	LL (mm/Leaf)	LW (mm/Leaf)
ZEA-1	2.0 ± 0.84 b,c	46.7 ± 10.7 c	5.4 ± 1.56 b,c	1097 ± 365 a,b,c	8.8 ± 3.38 b	26.7 ± 8.9 b	120 ± 83 b	0 d	13.8 ± 4.6 c	22.9 ± 4.6 a,b	17.0 ± 3.8 a,b
ZEA-2	2.0 ± 0.54 a,b,c	52.3 ± 11.0 b,c	6.5 ± 1.85 b	1422 ± 495 a	7.8 ± 2.76 c	25.1 ± 9.3 b	129 ± 84 b	72 ± 249 d	15.3 ± 3.8 b,c	25.3 ± 3.3 a	19.4 ± 3.0 a
BA-1	2.4 ± 0.60 a,b	34.0 ± 9.6 d	9.3 ± 2.95 a	1044 ± 446 b,c	2.0 ± 4.96 d	4.6 ± 11.4 c	24 ± 74 c	589 ± 229 c,d	17.2 ± 5.0 a,b	19.4 ± 4.1 b,c	12.6 ± 3.1 d
BA-2	2.4 ± 0.81 a,b	32.0 ± 7.3 d	9.7 ± 3.51 a	1148 ± 412 a,b	0 d	0 c	0 c	1069 ± 408 c	18.2 ± 6.1 a	16.1 ± 3.9 c	12.4 ± 4.2 d
TDZ-1	0 d	0 e	0 d	0 f	0 d	0 c	0 c	3943 ± 2074 a	0 e	0 d	0 e
TDZ-2	0 d	0 e	0 d	0 f	0 d	0 c	0 c	2439 ± 1046 b	0 e	0 d	0 e
MTP-1	2.4 ± 0.81 a,b	26.7 ± 6.5 d	6.5 ± 1.16 b	810 ± 193 c,d	0 d	0 c	0 c	168 ± 56 d	13.1 ± 2.4 c	16.9 ± 3.9 c	12.2 ± 2.6 d
MTP-2	2.5 ± 0.87 a	30.3 ± 10.7 d	6.8 ± 1.16 b	1064 ± 484 b,c	0 d	0 c	0 c	367 ± 149 d	13.6 ± 2.3 c	16.7 ± 5.8 c	13.1 ± 5.1 c,d
2iP-1	1.6 ± 0.58 c	58.2 ± 16.8 a,b	5.2 ± 1.79 b,c	1103 ± 447 a,b,c	11.8 ± 5.04 a	43.6 ± 17.6 a	239 ± 182 a	29 ± 147 d	12.2 ± 4.6 c	23.0 ± 7.6 a,b	18.6 ± 5.4 a
2iP-2	1.9 ± 0.33 b,c	65.6 ± 10.7 a	5.8 ± 1.96 b,c	1307 ± 410 a,b	10.6 ± 3.65 a,b	47.4 ± 16.0 a	183 ± 124 a,b	605 ± 284 c,d	12.9 ± 2.6 c	24.0 ± 2.8 a	15.2 ± 2.5 b,c
K	2.0 ± 0.20 a,b,c	34.5 ± 7.1 d	4.8 ± 1.36 c	697 ± 243 d	10.4 ± 3.40 a,b	48.0 ± 22.3 a	124 ± 65 b	0 d	9.4 ± 3.1 d	22.0 ± 6.6 a,b	15.7 ± 4.9 b,c

**Table 7:** Mean values of measured parameters with standard errors, effects of various cytokinins applied in the shoot multiplication medium on shoot growth and multiplication with 3% saccharose content. Different letters following the data indicate significantly (*p* < 0.05) different values between treatments according to ANOVA and Tukey tests (Abbreviations: SN: shoot number, SL: shoot length, NN: nodal number, SW: shoot weight, RN: root number, RL: root length, RW: root weight, CW: callus weight, LN: leaf number, LL: leaf length, LW: leaf width, ZEA: Zeatin, BA: Benzyladenine, TDZ: Thidiazuron, MTP: meta-Topolin, 2iP: N6-(2-Isopentenyl)adenine, K: control, cytokinins-1: 4.4 µM concentration, ytokinins-2: 13.2 µM concentration).

Cytokinin Content of the Media	SN (pcs/Explant)	SL (mm/Shoot)	NN (pcs/Shoot)	SW (mg/Shoot)	RN (pcs/Explant)	RL (mm/Explant)	RW (mg/Explant)	CW (mg/Explant)	LN (pcs/Shoot)	LL (mm/Leaf)	LW (mm/Leaf)
ZEA-1	2.2 ± 0.64 a,b,c	89.4 ± 19.4 a,b	7.8 ± 2.02 a	2250 ± 869 a	20.1 ± 6.5 b	143 ± 51 a	1293 ± 772 a,b	1487 ± 719 e	17.6 ± 4.7 b	23.2 ± 4.7 c	18.3 ± 3.9 b,c
ZEA-2	1.7 ± 0.46 d,e	83.8 ± 18.8 a,b	6.2 ± 1.22 b,c,d	1543 ± 466 c,d	9.0 ± 3.2 d	167 ± 38 a	1233 ± 642 a,b	2676 ± 763 c,d	12.6 ± 3.2 d,e	25.7 ± 3.1 b,c	17.2 ± 4.4 c,d
BA-1	2.5 ± 0.92 a,b,c	49.8 ± 9.6 c	7.8 ± 1.84 a	2073 ± 943 a,b	15.0 ± 7.7 c	104 ± 49 b	453 ± 409 c	3320 ± 622 c	17.9 ± 3.6 a,b	27.4 ± 5.6 b	15.0 ± 3.2 d
BA-2	2.0 ± 0.35 c,d	33.5 ± 7.8 d	5.4 ± 1.16 d	818 ± 267 e	1.3 ± 2.9 e	7 ± 15 c	13 ± 34 c	3354 ± 760 c	11.8 ± 2.5 d,e	14.6 ± 3.3 d	11.3 ± 2.3 e
TDZ-1	1.4 ± 0.51 e,f	9.6 ± 3.8 e	1.7 ± 0.80 e	276 ± 122 f	0 e	0 c	0 c	6284 ± 2088 a	3.0 ± 1.5 f	9.0 ± 3.7 e	3.7 ± 1.8 f
TDZ-2	1.0 ± 0.0 f	11.2 ± 4.6 e	1.0 ± 0.0 e	361 ± 136 f	0 e	0 c	0 c	4898 ± 1504 b	2.2 ± 1.3 f	5.1 ± 3.6 f	2.3 ± 1.3 f
MTP-1	2.7 ± 1.07 a,b	49.0 ± 17.0 c	8.0 ± 2.24 a	1957 ± 582 a,b,c	8.1 ± 6.0 d	31 ± 25 c	277 ± 361 c	1285 ± 670 e	20.5 ± 5.6 a	23.4 ± 3.6 c	19.7 ± 4.3 b,c
MTP-2	2.9 ± 0.88 a	41.3 ± 10.0 c,d	7.2 ± 2.95 a,b,c	1599 ± 655 b,c,d	0.1 ± 0.5 e	4 ± 12 c	1 ± 6 c	3164 ± 1007 c,d	16.1 ± 4.8 b,c	34.1 ± 6.5 a	19.0 ± 4.8 b,c
2iP-1	2.1 ± 0.78 c,d	94.6 ± 17.2 a	7.3 ± 1.77 a,b	2011 ± 742 a,b,c	16.4 ± 11.4 b,c	136 ± 80 a	1513 ± 1250 a	716 ± 484 e,f	14.6 ± 3.5 c,d	25.8 ± 5.2 b,c	20.9 ± 5.8 a,b
2iP-2	1.9 ± 0.52 d,e	82.0 ± 15.0 b	5.8 ± 1.34 c,d	1413 ± 375 d	9.6 ± 2.5 d	163 ± 40 a	1552 ± 702 a	2484 ± 558 d	11.4 ± 2.7 e	27.4 ± 3.6 b	17.7 ± 4.1 b,c,d
K	2.0 ± 0.0 c,d	91.3 ± 9.0 a,b	6.1 ± 0.93 b,c,d	1968 ± 462 a,b,c	30.9 ± 11.0 a	140 ± 35 a	907 ± 349 b	0 f	12.2 ± 1.9 d,e	29.3 ± 5.4 b	23.6 ± 5.0 a

**Table 8:** Mean values of measured parameters with standard errors of propagated shoots, on media containing different types of cytokinins in two concentrations, then rooted on media, complemented with or without NAA of rooted plantlets. Different letters following the data indicate significantly (*p* < 0.05) different values between treatments according to ANOVA and Tukey tests (Abbreviations: SN: shoot number, SL: shoot length, NN: nodal number, SW: shoot weight, RN: root number, RL: root length, RW: root weight, LN: leaf number, LL: leaf length, LW: leaf width, BA: Benzyladenine, MTP: meta-Topolin, NAA: 1-Naphthalene acetic acid).

Cytokinin in Multiplication Media	Rooting Media	SN (pcs/Plantlet)	SL (mm/Plantlet)	NN 8pcs/Plantlet	SW (mg/Plantlet)	RN (pcs/Plantlet)	RL (mm/Plantlet)	RW (mg/Plantlet)	LN (pcs/Plantlet)	LL (mm/Plantlet)	LW (mm/Plantlet)
BA	-	1.2 ± 0.09 a	34.0 ± 1.5 a	3.5 ± 0.18 b	446 ± 17 b	21.2 ± 0.7 a	33.4 ± 1.0 a	90 ± 6 c	7.8 ± 0.4 b	22.1 ± 0.7 a	14.4 ± 0.45 a,b
	NAA	1.2 ± 0.12 a	31.7 ± 1.6 a,b	4.2 ± 0.29 a	562 ± 65 a	21.6 ± 0.8 a	29.1 ± 1.2 b	202 ± 23 a	10.6 ± 1.4 a	20.8 ± 0.9 a	15.7 ± 0.8 a
MTP	-	1.1 ± 0.06 a	29.2 ± 1.7 b	3.2 ± 0.17 b	431 ± 31 b	20.1 ± 1.1 a	35.9 ± 1.7 a	146 ± 16 b	7.8 ± 0.5 b	19.8 ± 1.1 a	12.7 ± 0.8 b
	NAA	1.2 ± 0.09 a	31.0 ± 1.4 a,b	4.0±0.15 a	482 ± 25 a,b	22.1 ± 0.8 a	27.7 ± 0.9 b	193 ± 14 a	8.4 ± 0.3 b	22.1 ± 0.6 a	15.5 ± 0.5 a

**Table 9:** Mean values of measured parameters with standard errors of hardened plants, multiplied on media containing different types of cytokinins in two concentrations, then rooted on media, complemented with or without NAA. Different letters following the data indicate significantly (*p* < 0.05) different values between treatments according to ANOVA and Tukey tests. (Abbreviations: B: branching value, SL: shoot length, SN: shoot number, SW: shoot weight, FB: flower bud number, 1LL: first leaf length, 1LW: first leaf width, 2LL: second leaf length, 2LW: second leaf width, RL: root length, RW: root weight, BA: Benzyladenine, MTP: meta-Topolin, NAA: 1-Naphthalene acetic acid).

Mult. Med.	Rooting	B (Shoot/Plat)	SL (mm/Plant)	SN (pcs/Plant)	SW (mg/Plant)	FB (pcs/Plant)	1LL (mm/Leaf)	1LW (mm/Leaf)	2LL (mm/Leaf)	2LW (mm/Leaf)	RL (mm/Plant)	RW (mg/Plant)
BA	-	1.6 ± 0.09 b	88.7 ± 2.6 a	1.8 ± 0.18 a	492 ± 0.20 a	1.2 ± 0.09 a	73.7 ± 2.3 a	45.3 ± 1.5 a	74.1 ± 2.3 a	45.5 ± 1.4 a	91.6 ± 8.0 a	615 ± 75 a
	NAA	1.8 ± 0.07 a	76.2 ± 3.7 b	1.3 ± 0.12 a	378 ± 0.29 b	1.0 ± 0.11 a	75.5 ± 3.9 a	44.9 ± 2.6 a	74.8 ± 4.1 a	45.2 ± 2.4 a	81.3 ± 7.7 a	477 ± 56 a
MTP	-	1.6 ± 0.08 a,b	86.4 ± 2.0 a	1.8 ± 0.20 a	427 ± 0.16 b	1.2 ± 0.11 a	72.1 ± 2.5 a	44.5 ± 1.7 a	69.5 ± 2.7 a	44.6 ± 1.7 a	92.2 ± 8.1 a	615 ± 83 a
	NAA	1.6 ± 0.09 b	75.9 ± 3.9 b	1.8 ± 0.18 a	419 ± 0.20 b	1.0 ± 0.07 a	76.9 ± 2.8 a	48.6 ± 1.9 a	77.7 ± 3.0 a	47.3 ± 1.9 a	102.1 ± 9.6 a	663 ± 80 a

## Data Availability

The original contributions presented in this study are included in the article. Further inquiries can be directed to the corresponding author.

## Abbreviations

MS	Murashige and Skoog
CK	Cytokinin
IAA	Indole-3-acetic acid
KIN	kinetin
BA	Benzyladenine
AgNO_3_	Silver nitrate
IBA	Indole-3-butyric acid
2,4-D	2,4-Dichlorophenoxyacetic acid
TDZ	Thidiazuron
NAA	1-Naphthalene acetic acid
AA	Ascorbic acid
PGR	Plant Growth Regulator
HgCl_2_	Mercuric II chloride
MWP	McCown Woody Plant
ZEA	Zeatin
2iP	N6-(2-Isopentenyl)adenine
MTP	meta-Topolin
NaOCl	Sodium hypochlorite
SDW	Sterile deionized water
